# Effects of Cholesterol and Solution pH on Daptomycin-bilayer Interactions

**DOI:** 10.1007/s00232-026-00400-8

**Published:** 2026-08-29

**Authors:** Samuel R. Pennock, Maggie Cortes, Megha D. Salecha, Neil X. Sevilla, Nicole R. Rementer, Kevin Rana, Luke C. Hicks, Emmaleigh Fox, Ahmed Grine, Patience S. Hammel, Timothy D. Vaden, Benjamin R. Carone, Gregory A. Caputo

**Affiliations:** 1 Department of Chemistry & Biochemistry, Rowan University, Glassboro, NJ USA; 2 Department of Biological & Biomedical Science, Rowan University, Glassboro, NJ USA

**Keywords:** Daptomycin, Model membranes, Fluorescence, Antimicrobial peptides

## Abstract

The global challenge of increasing antibiotic resistance development has necessitated the continued development and investigation of novel and existing antimicrobials. Daptomycin is a cyclic lipopeptide isolated from *Streptomyces roseosporus*, which has been clinically approved to treat a variety of gram-positive infections. Daptomycin is a membrane-active antimicrobial peptide that requires Ca^2+^ ions and phosphatidylglycerol lipids to exert its antimicrobial activity. To better understand the mechanism of action at the biophysical level, daptomycin interactions with bilayers and bacterial membranes were evaluated across different solution pH values and by varying cholesterol content in the model membranes. The results demonstrate that pH 5–8 does not affect daptomycin binding to model membranes or its structural rearrangement upon binding to the bilayer. Similarly, including cholesterol up to 30 mol% in the bilayers did not affect daptomycin binding, with an apparent binding affinity of ~ 1 µM under these conditions. Fluorescence quenching experiments showed that Ca^2+^ binding to daptomycin induced a small but significant shift in the position of Trp in the bilayer, becoming more deeply inserted when calcium was present. Bacterial membrane permeabilization assays confirmed that daptomycin forms stable pores in bacterial membranes large enough to cause leakage of the chromogenic substrate, o-nitrophenyl-β-D-galactopyranoside (ONPG). The data demonstrate a remarkable robustness of daptomycin interactions with lipid bilayers.

**Graphical Abstract:**

**Figure:**
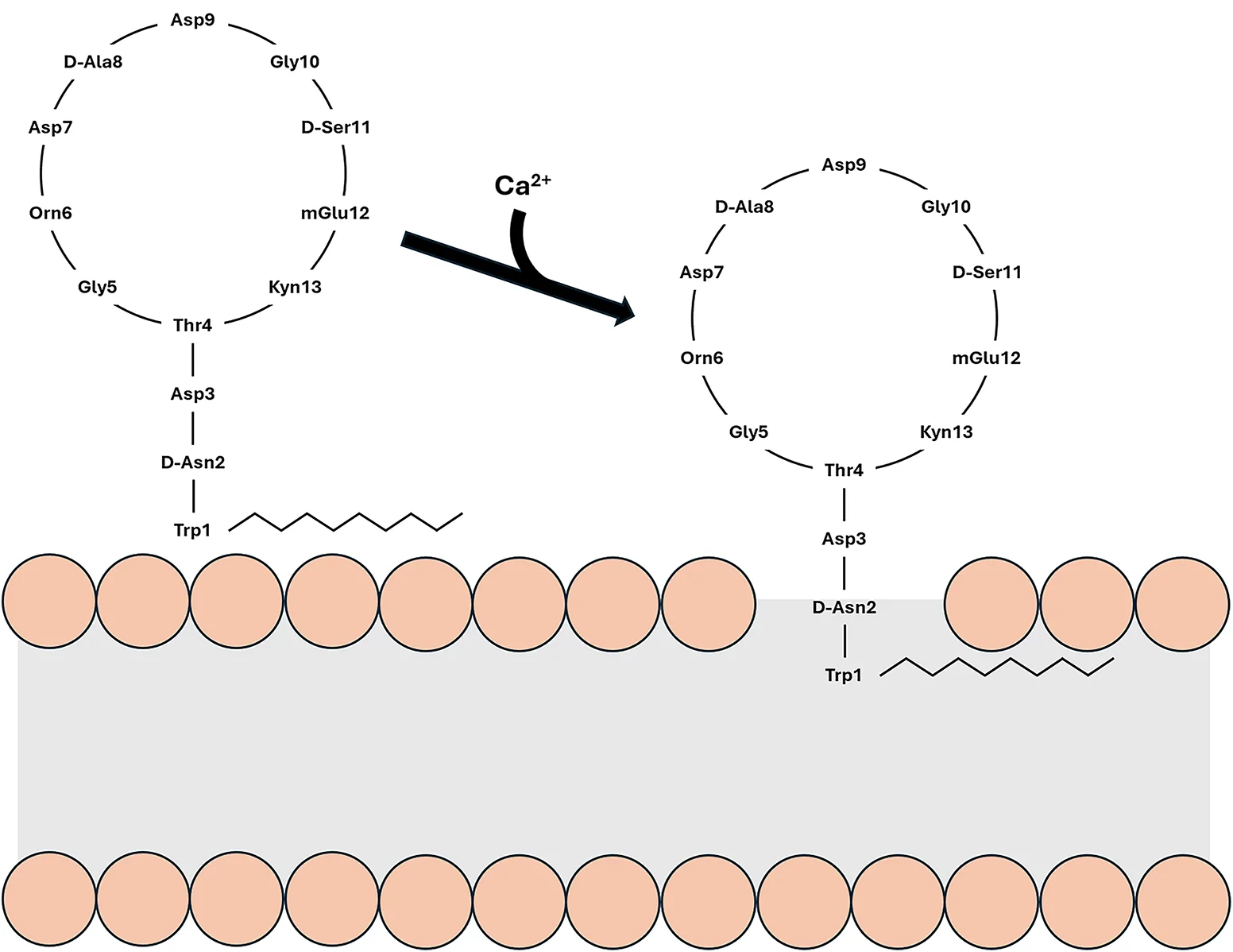

**Supplementary Information:**

The online version contains supplementary material available at https://doi.org/10.1007/s00232-026-00400-8.

## Introduction

Antimicrobial resistance (AMR) has been identified as one of the most pressing threats to human health for the coming decades. In 2019, healthcare data indicate that ~ 1.2 million deaths were directly attributable to antimicrobial-resistant infections and indirectly related to ~ 4.2 million more deaths (Antimicrobial Resistance 2022; Ho et al. 2025; Salam et al. 2023). Based on these data, the predicted number of deaths directly linked to AMR is estimated to rise to ~ 10 million annually (Antimicrobial Resistance 2022; Salam et al. 2023). The AMR crisis is directly linked to many other conditions and comorbidities, and exhibited a dramatic uptick during the COVID-19 pandemic due to increased rates of hospitalization (Lai et al. 2021; Langford et al. 2023). AMR occurs when bacteria develop resistance to an antimicrobial agent through a number of mechanisms including random mutations in the bacterial genome, selection pressure exacerbated by improper use and/or prescription of antibiotics, horizontal gene transfer, and other mechanisms (MacLean and San Millan 2019; Richardson 2017; Smith et al. 2015).

The AMR crisis has spurred significant interest in the development of novel antimicrobials, as well as improvements in drug delivery and clinical practices, both in prescribing antibiotics and in treating infections. Many groups have pursued the development of antibacterial compounds beyond the traditional small organic-molecule framework. These include expanded study of antibacterial natural products, phage therapy, metal ions and metal nanoparticles, polymers, antimicrobial peptides (AMPs), and peptidomimetics (Alaoui Mdarhri et al. 2022; Ghosh et al. 2019; Kuroda and Caputo 2013). However, few of these molecules have cleared clinical trials to date. That said, there has been only incremental success in translating naturally derived antimicrobial peptides into clinical use. Importantly, the majority of successful cases have involved lipopeptides such as polymyxins and daptomycin (Al Musaimi 2025; Lv et al. 2026).

**Fig. 1:**
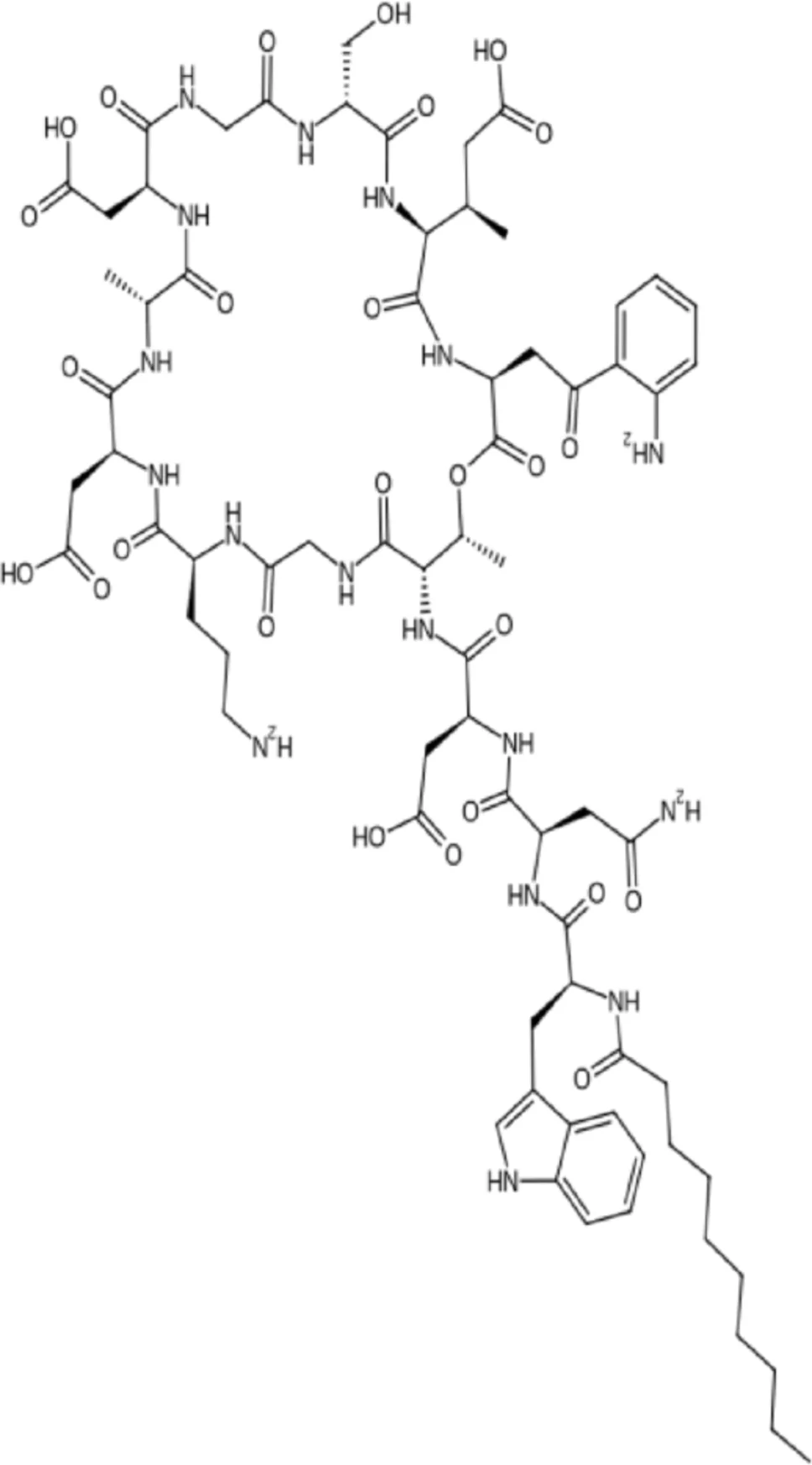
Structure of daptomycin. decanoyl-L-Trp-D-Asn-L-Asp-L-Thr*-L-Gly-L-Orn-L-Asp-D-Ala-L-Asp-L-Gly-D-Ser-L-mGlu-L-Kyn*. ‘*’ denotes a lactone bond between the carboxylic group of L-Kyn13 and the β-hydroxyl group of L- Thr4, forming the macrocycle

Daptomycin is an FDA-approved, cyclic lipopeptide antibiotic originally isolated from *Streptomyces roseosporus* (Fig. 1) (Eisenstein et al. 2010). The structure consists of 13 amino acids, including multiple non-canonical residues (D-Asn, D-Ala, ornithine, D-Ser, 3-methyl-glutamate, kynurenine) as well as a C10 decanoyl modification on Trp1. The macrocycle in the daptomycin structure is formed from 10 amino acids linked by a lactone formed between the backbone carboxyl of kynurenine (Kyn13) and the side chain alcohol of Thr3. The daptomycin mechanism of action first involves binding phosphatidylglycerol headgroups in the target membrane, which are abundant in the membranes of Gram-positive bacteria, which is significantly stabilized through a tripartite interaction with Ca^2+^ ions. Once bound, the daptomycin C10 chain inserts into the bacterial membrane, where it disrupts membrane integrity and has also been implicated in disrupting protein transport across the membrane (Buttress et al. 2025; Huang 2020).

Despite a well-studied mechanism of action, several questions remain regarding the biochemical and biophysical properties of daptomycin. The fact that daptomycin is effective only against gram-positive organisms, despite gram-negative bacteria also containing significant amounts of PG in their membranes, has been a lingering question (Bogdanov et al. 2020). Similarly, the nature of the daptomycin oligomer in the membrane as well as the pore conformation/mechanism are not fully understood.

We sought to gain a deeper understanding of daptomycin interactions with lipid membranes by evaluating its binding to and topography within lipid bilayers across various pH conditions and lipid bilayer compositions. The intrinsic Trp fluorescence in daptomycin was utilized, allowing for an environmentally sensitive probe that reports on both bilayer interactions and changes in bound conformations, and it was utilized in quenching assays to evaluate these changes. In the first set of experiments, we investigated the impact of incorporating cholesterol into the bilayer, which is not present in bacterial membranes but is found in host mammalian membranes. The second set of experiments built on these and further evaluated the effect of solution pH on daptomycin interactions with model lipid vesicles. The results show the remarkable robustness of the daptomycin molecule in retaining activity under different conditions as well as demonstrating that the Trp position with respect to the membrane becomes somewhat more deeply inserted in the bilayer upon binding Ca^2+^.

## Experimental Methods

### Materials

The following lipids: 1-palmitoyl-2-oleoyl-glycero-3-phosphocholine (POPC) (Item # 850457), 1-palmitoyl-2-oleoyl-sn-glycero-3-phospho-(1’-rac-glycerol) (POPG) > 99% pure (Item# 840457), 10-doxyl nonadecane (810590O), and cholesterol (700100), were purchased as powder from Avanti Research (Alabaster AL). Lipids were stored at -20 °C until needed. Powdered daptomycin 99.73% pure (Cat. No.: HY-B0108) was purchased from MCE (MedChemExpress, Monmouth Junction, NJ USA) and stored at -20 °C until needed. All other salts and buffers were from VWR (Radnor, PA USA).

### Buffer Preparation

Eight Buffers were prepared to examine the interactions between daptomycin and lipid vesicles at four different pH values ± excess Ca^2+^. The following buffers were prepared for the different experiments: 20 mM sodium acetate (NaOAc), 150 mM NaCl, pH 4.0; 20 mM sodium acetate (NaOAc), 140 mM NaCl, 5 mM CaCl_2_, pH 4.0; 20 mM sodium acetate (NaOAc), 150 mM NaCl, pH 5.0; 20 mM sodium acetate (NaOAc), 140 mM NaCl, 5 mM CaCl_2_, pH 5.0; 20 mM Tris, 150 mM NaCl, pH 7.0; 20 mM Tris, 140 mM NaCl, 5 mM CaCl_2_, pH 7.0; 20 mM Tris, 150 mM NaCl, pH 8.0; 20 mM Tris, 140 mM NaCl, 5 mM CaCl_2_, pH 8.0. Once prepared, buffers were adjusted to the correct pH using dilute HCl or NaOH. Once pH was corrected, buffers were aseptically filter-sterilized through a 0.22 µM polycarbonate filter into autoclaved glassware and stored at 4 °C until needed.

### Lipid Vesicle Preparation

Lipids purchased from Avanti Research (POPC, POPG, and cholesterol) were dissolved in chloroform to the final concentrations of 12.97, 13.16, and 10.13 mM, respectively (based on dissolving 100 mg of lipid to a final volume of 10 mL) and stored in scintillation vials sealed with Teflon tape at -20 °C until needed. To prepare the lipid vesicles, dissolved lipid stocks were allowed to reach room temperature before opening to prevent water contamination from condensation. Once at room temperature, lipid stocks were combined, as specified in Table 1, in glass test tubes using a positive-displacement pipette, such that the total lipid concentration when suspended in 1.5 mL of buffer was 1.0 mM. Once combined in a test tube, chloroform was evaporated under a drying manifold of inert N_2_(*g*) at ~ 2 PSI for 20 min, or until all chloroform was evaporated, leaving a lipid film at the bottom of the test tube. The test tube was then stored in a vacuum desiccator for at least 1 h but no more than 24 h to ensure the lipid film was dry and free of chloroform. Following desiccation, the lipid film was resuspended in 1.5 mL of the desired buffer, yielding an opaque, cloudy solution. The solution was then transferred to a 13 × 100 Pyrex test tube and sealed before being placed in a high-power water bath sonicator for 20 min to ensure that all spontaneously formed multilamellar vesicles were converted to unilamellar vesicles. The size of the unilamellar vesicles was confirmed via dynamic light scattering (DLS) using a Malvern Zetasizer Pro, and representative vesicle size data is shown in supplementary Table S1. Lipid vesicles were used immediately after preparation and stored at 4 °C for no longer than one hour.

**Table 1:** Lipid Vesicle Compositions

**Lipid vesicle #**	**[Lipid] (mM)**		
	**POPC**	**POPG**	**Cholesterol**
LV1	0.75	0.25	0
LV2	0.7	0.25	0.05
LV3	0.6	0.25	0.15
LV4	0.5	0.25	0.25
LV5	0.45	0.25	0.30

### Daptomycin Preparation

Daptomycin (MW = 1,619.709 g/mol) was aseptically dissolved in distilled, deionized water to a final concentration of 1.0 mg/mL (≈ 617.4 µM) and stored in 1.0 mL aliquots at -20 °C until needed. Based on preliminary spectroscopic data, the ideal working concentration of daptomycin in solution was 20 µM for fluorescence assays and 100 µM for absorbance or circular dichroism assays. Daptomycin was aseptically diluted to the appropriate working concentration in the desired buffer immediately before use.

### Absorbance and Circular Dichroism Spectroscopy

Daptomycin was diluted to 100 µM in the desired buffer and augmented with 500 µM lipid vesicles if required. For absorbance spectra, 1.0 mL was transferred to a 1.4 mL semi-micro quartz cuvette (path length = 1.0 cm) and the absorbance (A.U.) was measured from 200 to 500 nm in triplicate by a VWR UV/Vis – 100PC spectrophotometer. For circular dichroism (CD) spectroscopy, 0.4 mL was transferred to a low-volume quartz cuvette (path length 10 mm), and molar ellipticity (θ) (degrees·cm²/dmol) was recorded from 260 –190 nm with a Jasco J-1500 CD spectropolarimeter. For CD samples with and without lipid vesicles, 64 and 8 accumulations were taken, respectively, at a continuous scanning speed of 50 nm/min.

### Daptomycin Binding to Lipid Vesicles

Daptomycin was diluted to 20 µM in the desired buffer, and 1.0 mL was transferred to a quartz cuvette. Fluorescence spectra of Daptomycin were recorded in triplicate with a Horiba FluoroMax-4 spectrofluorometer maintained at a constant temperature of 20 °C by a Quantum Northwest TC1 Temperature Controller and Koolance Liquid Cooling system. Due to the molecular proximity of Trp-1(λ_ex_ = 280 nm, λ_em_ = 350 nm) to Kyn-13 (λ_ex_ = 365 nm, λ_em_. = 480 nm) in daptomycin, Förster resonance energy transfer (FRET) can and will occur (Lee et al. 2017). Preliminary experiments determined that the ideal fluorescence conditions to observe the blue shift of Trp fluorescence in daptomycin are λ_ex_. = 280 nm and λ_em_. = 400–520 nm. The fluorescence spectrum of daptomycin was recorded as precise amounts of lipid vesicle were thoroughly mixed into the solution containing 20 µM daptomycin. At each concentration of lipid vesicle, the barycenter (the weighted average wavelength of emission) of the emission spectra of daptomycin could be determined by the following equation:

1 $$\:B\left(nm\right)=\frac{\sum\:\left(i*\lambda\:\right)}{\sum\:\left(i\right)}$$

Where ‘*i*’ is the intensity (counts per second (CPS)), and ‘λ’ is the wavelength at which the intensity was recorded. The normalized fraction bound (*F*_*B*_) of daptomycin at a given condition, ‘*x*’, was then extrapolated from the barycenter by the following equation:

2 $$\:{F}_{B}\left(x\right)=\frac{\left(B\right(x)-{B}_{min})}{({B}_{max}-{B}_{min})}$$

The relative binding affinity (*k*_*d*_) of daptomycin for each lipid vesicle could be determined from the plot of *F*_*B*_ versus the concentration of lipid vesicle (corrected for dilution factor) by fitting the plot in OriginPro to the following modified Langmuir adsorption model:

3 $$\:{F}_{B}\left(x\right)=\frac{\left[LV\right]\left(x\right)}{{k}_{d}+\left[LV\right]\left(x\right)}$$

As this system does not fully meet the assumptions of the Langmuir adsorption model, any fitted parameters (*k*_*d*_) should be interpreted as representative rather than an exact physical model (Latour 2015).

### Fluorescence Quenching – Aqueous

Acrylamide was dissolved to a concentration of 4.0 M in distilled deionized water. Daptomycin and lipid vesicles were diluted from the stock concentrations to 20 µM and 50 µM, respectively, in the desired buffer, and 1.0 mL was transferred to a 1.5 mL quartz cuvette. A ratio of 20 µM daptomycin to 50 µM lipid vesicles was used, as experimental testing showed that, under all conditions and in the presence of excess Ca²⁺, daptomycin was fully membrane-associated at this concentration. Single-point fluorescence intensity (CPS), λ_ex_. = 295 nm, λ_em_. = 465, was recorded in triplicate with a Horiba FluoroMax-4 spectrofluorometer maintained at a constant temperature of 20 °C by a Quantum Northwest TC1 Temperature Controller and Koolance Liquid Cooling system. The single-point emission in the absence of acrylamide quencher (*F*_*0*_) was recorded. Then, 10 µL aliquots of 4.0 M acrylamide were added sequentially and thoroughly mixed into the sample, up to a total added volume of 50 µL, with single-point emission spectra recorded after each addition. The ratio of the fluorescence intensity in the absence of quencher (*F*_*0*_) to the intensity at each concentration of quencher (*F*) was graphed against the concentration of acrylamide (M). The resulting plot followed the Stern-Volmer equation:

4 $$\:\frac{{F}_{0}}{F}=1+\:{K}_{sv}\left[X\right]$$

Where ‘*X*’ is the concentration of quencher (acrylamide) and ‘*K*_*sv*_’ is the Stern-Volmer constant, which quantifies the susceptibility of the fluorophore to the quencher and is analogous to the slope of a linear regression (Lehrer 1971).

### Fluorescence Quenching – Bilayer Associated

10-doxyl nonadecane (10-DN) is a highly hydrophobic quencher of Trp fluorescence that partitions into the center of lipid bilayers (Caputo and London 2003). 10-DN (MW = 368.62 g/mol) was dissolved in chloroform to a final concentration of 1.0 mg/mL (≈ 2.71 mM). 10-DN was incorporated into the lipid vesicle preparation as an additional lipid. Lipid vesicle composition for bilayer-associated fluorescence quenching is outlined in Table 2. All other methods for preparing lipid vesicles were followed as previously described (Caputo and London 2019). Dynamic light scattering (DLS) with a Malvern Zetasizer Pro confirmed that the resulting lipid vesicles had a diameter of ~ 122 nm. Once synthesized, lipid vesicles were diluted to 50 µM with 20 µM daptomycin in the desired buffer. A ratio of 20 µM daptomycin to 50 µM lipid vesicles was used, as experimental testing showed that, under all conditions and in the presence of excess Ca²⁺, daptomycin was fully membrane-associated at this concentration. Single-point fluorescence intensity (CPS), λ_ex_. = 280 nm, λ_em_. = 465, was recorded in triplicate with a Horiba FluoroMax-4 spectrofluorometer maintained at a constant temperature of 20 °C by a Quantum Northwest TC1 Temperature Controller and Koolance Liquid Cooling system. The ratio of single-point fluorescence intensity of the unquenched bilayer (*F*_*0*_) to the quenched bilayer (*F*_*q*_) was subsequently recorded.

**Table 2:** Lipid Vesicle Compositions for Quenching Experiments

**Lipid vesicle #**	**[Lipid] (mM)**			
	**POPC**	**POPG**	**Cholesterol**	**10-DN**
LV1	0.75	0.25	0	0
LV1*	0.65	0.25	0	0.1
LV5	0.45	0.25	0.3	0
LV5*	0.35	0.25	0.3	0.1

* indicates vesicles containing 10-DN

### ζ-Potential Measurements

Lipid vesicles were diluted in the desired buffer to a concentration of 100 µM, with daptomycin concentrations of 0.0, 5.0, 15.0, and 25.0 µM, and allowed to equilibrate for 5 min at room temperature. Each sample was transferred to a Malvern Zetasizer Nano Series Disposable folded capillary cell (DTS1070) and the ζ-potential (mV) of the lipid vesicle was recorded by a Malvern Zetasizer Pro DLS spectrophotometer.

### Antimicrobial Assays

*Acinetobacter baumannii* (ATCC: 19606), *Bacillus subtilis* (ATCC 6051), *Enterococcus faecalis* (ATCC 29212), *Escherichia coli* (CGSC#:5165), *Klebsiella pneumoniae* (ATCC 700603), *Staphylococcus aureus* (ATCC: 35556), *Staphylococcus epidermidis* (ATCC 14990), and *Streptococcus salivarius* (Carolina 155640 A) were stored as cold stocks at -80 °C. Minimal inhibitory concentration (MIC) and minimal bactericidal concentration (MBC) assays were performed as previously described (Hitchner et al. 2021). Briefly, overnight cultures were aseptically created from isolated colonies on Mueller-Hinton (MH) agar into MH broth and incubated at 37 °C and 220 RPM for 18 h, then diluted 1:200 in fresh MH broth and incubated at 37 °C and 220 RPM. When the OD600 of the cultures reached 0.2–0.5, bacteria were again diluted in fresh MH broth at the desired pH (4.0, 5.0, 7.0, and 8.0) such that the final cell density of bacteria in the treatment wells was 5 × 10^5^ cells/mL and mixed with serial dilutions of daptomycin or control compounds in a 96-well plate. The plate was incubated for 18 h at 37 °C. OD_600_ was measured using a Molecular Devices SpectraMax M5 multichannel spectrophotometer to assess bacterial growth. As described by Mavridou and coworkers, the MIC values represent the consensus, not the technical average, of the experiments (Kaderabkova et al. 2024). To determine minimal bactericidal concentration (MBC), 1.0 µL of each well was subsequently plated on an MH agar plate and incubated for an additional 18–24 h at 37 °C, after which bacterial growth was analyzed visually. All experiments were performed in triplicate from independent samples.

### Bacterial Membrane Permeabilization

Propidium iodide (PI, 3,8-Diamino-5-[3-(diethylmethylammonio)propyl]-6-phenylphenanthridinium diiodide) was dissolved to a stock concentration of 1 mg/mL (≈ 1.5 mM). The concentration of PI was confirmed using the Beer-Lambert law (ε_287nm_ = 55,700 M^− 1^ cm^− 1^), and the stock was stored at 4 °C in the absence of light until needed. Overnight cultures of *Staphylococcus aureus* (ATCC: 35556) were prepared in MH broth and incubated at 37 °C and 220 RPM for 18 h and subsequently diluted 1:200 in fresh MH broth and incubated at 37 °C and 220 RPM until the OD_600_ of the cultures reached 0.2–0.5. The cultures were then pelleted at 2,500 RPM in a benchtop clinical centrifuge for 15 min; the media was decanted, and the pellet was resuspended in pH-adjusted MH media to an OD_600_ of 0.2 A.U. The bacteria were then combined in a 96-well plate with serial dilutions of daptomycin or melittin (positive control) and a working concentration of 70 µM PI. At 0, 30, and 60 min after addition of PI the fluorescence emission was recorded with a BD FACSCelesta flow cytometer (Ex: 488 nm laser, Em: 575 filter.) to determine membrane permeabilization. All experiments were performed in triplicate from independent samples.

### Gram-positive Membrane Permeabilization (β-Galactosidase Assay)

An isolated colony of *S. aureus* A322 (O’Neill et al. 2004) was incubated overnight (37 °C, 220 RPM, 18 h) in Luria-Bertani (LB) broth augmented with 50 µg/mL chloramphenicol. The S. aureus culture was then diluted 1:200 into fresh LB broth augmented with 50 µg/mL chloramphenicol and 1 mM isopropyl β-D-1-thiogalactopyranoside (IPTG) and incubated (37 °C, 220 RPM) until an OD_600_ of 0.5 A.U. was reached. Antimicrobial peptides were suspended to a concentration of 150 µM in a 50:50 H2O: EtOH solution and serially diluted in 0.01% acetic acid on a template plate. On a clear flat-bottom 96-well plate, 45 µL of phosphate buffer supplemented with the reducing agent β-mercaptoethanol, 35 µL of *S. aureus* culture, 10 µL of the appropriate antimicrobial peptide treatment, and 15 µL of 4 mg/mL o-nitrophenyl-β-D-galactopyranoside (ONPG) were combined. The absorbance at 420 nm of each well was immediately monitored every 5 min for 90 min using a Molecular Devices – SpectraMax M5 multichannel plate reader. All antimicrobial peptides and treatment concentrations were performed in triplicate.

## Results

### Daptomycin Activity

To evaluate the functionality of daptomycin under the conditions tested, antimicrobial activity was determined against a panel of bacteria at different pH values. Antimicrobial activity was evaluated by determining the minimal inhibitory concentration (MIC) and the minimal bactericidal concentration (MBC) against both Gram-positive and Gram-negative bacteria. Briefly, these experiments expose the bacterial cultures to a range of concentrations of the antimicrobial compound to determine the lowest concentration required to inhibit growth in an overnight liquid culture (MIC), and subsequently those cultures are assayed for surviving bacteria by the ability to grow on antibiotic-free solid media (MBC). In these experiments, growth media were pH-adjusted prior to the MIC assay and addition of daptomycin. The data is shown in Table 3. Notably, none of the bacteria grew in pH 4 media, even in the complete absence of daptomycin, denoted as n.g. (no growth). However, all bacteria tolerated media at pH 5, 7, and 8 well and grew consistently. The MIC values were similar in pH 5, 7, and 8 media, with a 2x reduction in efficacy at pH 5 against gram-positive bacteria. Consistent with prior results, daptomycin was completely ineffective against gram-negative strains at pH 7, and this trend held true at pH 5 and 8.

**Table 3:** Minimal Inhibitory Concentration^a^ (µg/mL)

**Bacteria**		**pH 4.0**	**pH 5.0**	**pH 7.0**	**pH 8.0**
**Gram-positive**	*B. subtilis*	NG^b^	3	3	3
	*E. faecalis*		12	6	6
	*S. aureus*		12	6	6
	*S. epidermidis*		12	6	6
	*S. salivarius*		12	6	6
**Gram-negative**	*A. baumannii*		> 110	> 110	> 110
	*E. coli*		> 110	> 110	> 110
	*K. pneumoniae*		> 110	> 110	> 110

a: Minimal Bactericidal Concentrations (MBC) were identical to MIC values

b: NG = no growth of bacteria in untreated controls

### Binding to Vesicles

**Fig. 2:**
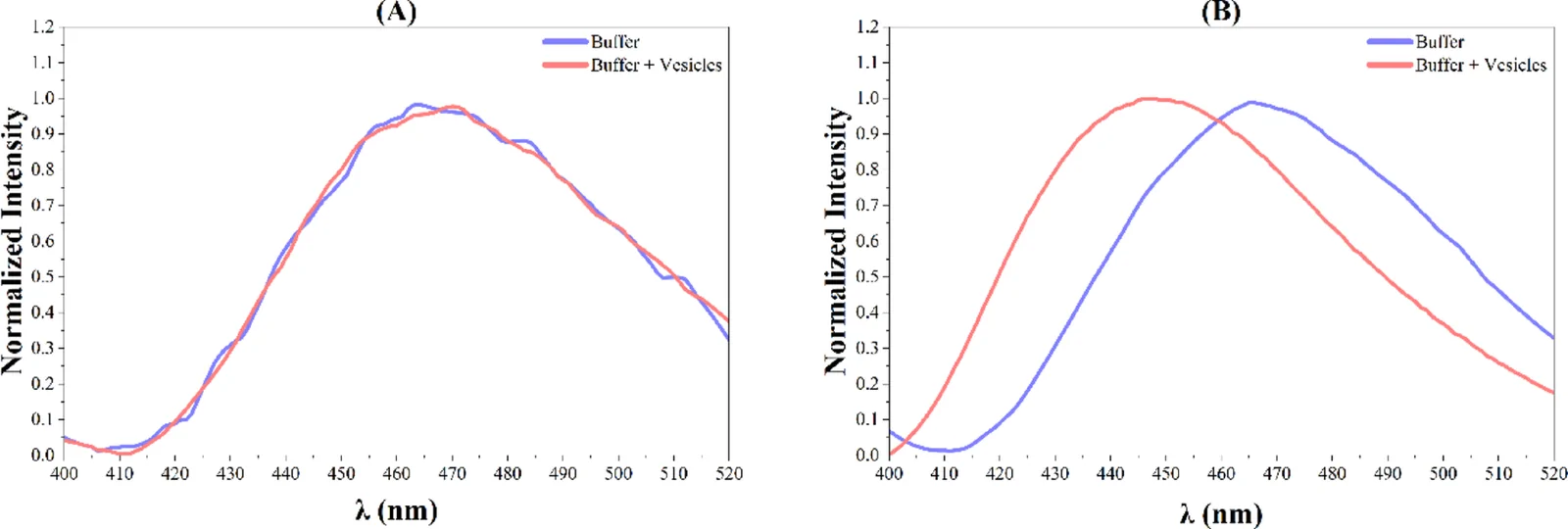
Representative spectra of daptomycin under various conditions. Normalized spectra of 20 µM daptomycin ± 50 µM lipid vesicle (75% POPC: 25% POPG) in buffer (20 mM Tris, pH 7.0) with **(A)** 150 mM NaCl and 0.0 mM CaCl_2_ or **(B)** 140 mM NaCl and 5.0 mM CaCl_2_

Daptomycin interactions with the model membranes were evaluated using fluorescence emission spectroscopy due to the environmental sensitivity of the emission spectrum of Trp. Notably, in daptomycin, the emission spectrum results from Trp undergoing FRET to the non-canonical amino acid kynurenine, yielding an emission centered at ~ 470 nm. Nonetheless, this emission spectrum responds similarly to Trp, exhibiting a significant blue shift when the fluorophore enters a more non-polar environment, such as when interacting with the lipid bilayer. Representative spectra are shown in Fig. 2, with additional plots demonstrating the shifts in both barycenter and intensity over the full titration shown in Supplemental Figures S1 & S2. These spectra demonstrate the dramatic blue-shift of the emission spectrum when both calcium and lipid vesicles are present. Notably, there is no discernible shift in the spectrum if only calcium or vesicles are present.

**Fig. 3:**
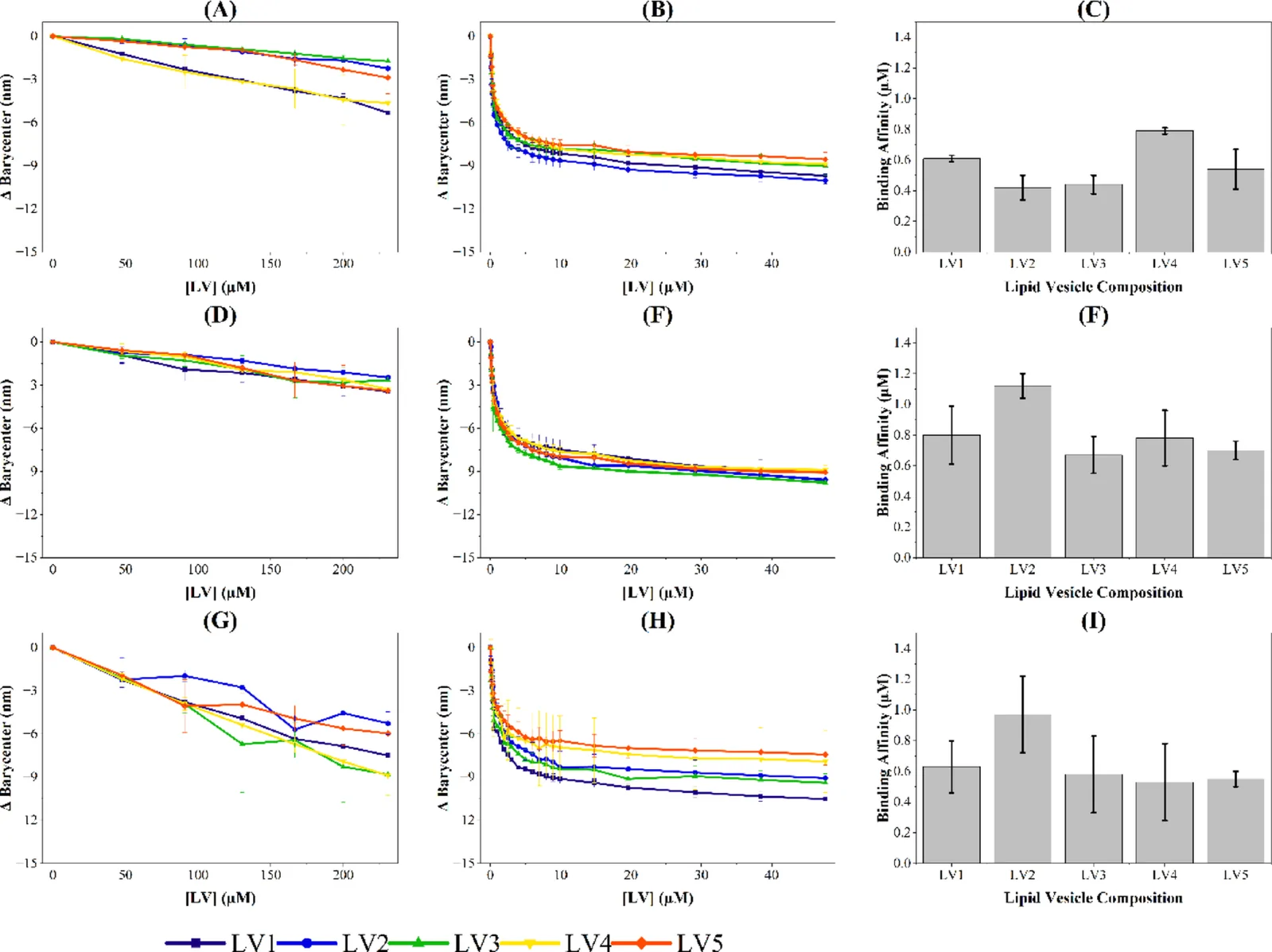
Affinity of daptomycin for lipid vesicles with varying [cholesterol] at three pHs (top row pH 5.0, middle row pH 7.0, bottom row pH 8.0). The blueshift in fluorescence (λ_ex_. = 280 nm, λ_em_. = 400–520 nm) barycenter of Trp-1 and Kyn-13 residues in daptomycin was monitored as lipid vesicles #1–5 were titrated into solution in either the absence of Ca^2+^ (first column) or the presence of 5.0 mM Ca^2+^ (second column). From the shift in barycenter, binding curves were extrapolated, and the binding affinity of *halo*-Ca^2+^-daptomycin for lipid vesicles under each condition was calculated (third column)

The binding experiments evaluated the interaction of daptomycin with lipid vesicles containing varying amounts of cholesterol in the bilayer at different pH values. Importantly, the presence of PG lipids has been shown to be critical for daptomycin interactions with lipid bilayers and subsequent antimicrobial activity. Here, vesicles were prepared with a constant amount of POPG (25 mol%) with the remaining 75 mol% composed of POPC and cholesterol, where the individual amounts were varied to allow for increasing cholesterol content in the bilayer. The data are shown in Fig. 3, with binding in the absence of calcium in the first column, in the presence of 5 mM CaCl_2_ in the second column, and the calculated apparent K_d_ values in the right column. Fractional occupancy curves with binding fits are shown in the supplemental data Figure S3. The rows in Fig. 3 represent experiments carried out at pH 5 (top row), pH 7 (middle row), and pH 8 (bottom row). These data indicate that while calcium has a dramatic effect on binding to the lipid vesicle, the inclusion of cholesterol does not appear to alter binding affinity. These results are consistent with the model in which the daptomycin peptide macrocycle binds to the bilayer surface through electrostatic interactions and only the C10 tail inserts into the bilayer. Similar to the results for the cholesterol experiments, environmental pH did not appear to affect daptomycin binding affinity for bilayers to a significant extent. This result is somewhat surprising in that at the lowest pH tested, this is near the pK_a_ of several of the carboxylates on daptomycin, which are involved in calcium binding. Nonetheless, it does not appear pH influences daptomycin bilayer interactions over a range of physiologically relevant pH values as judged by the K_d_ values calculated in Fig. 3C and F, and 3I.

**Fig. 4:**
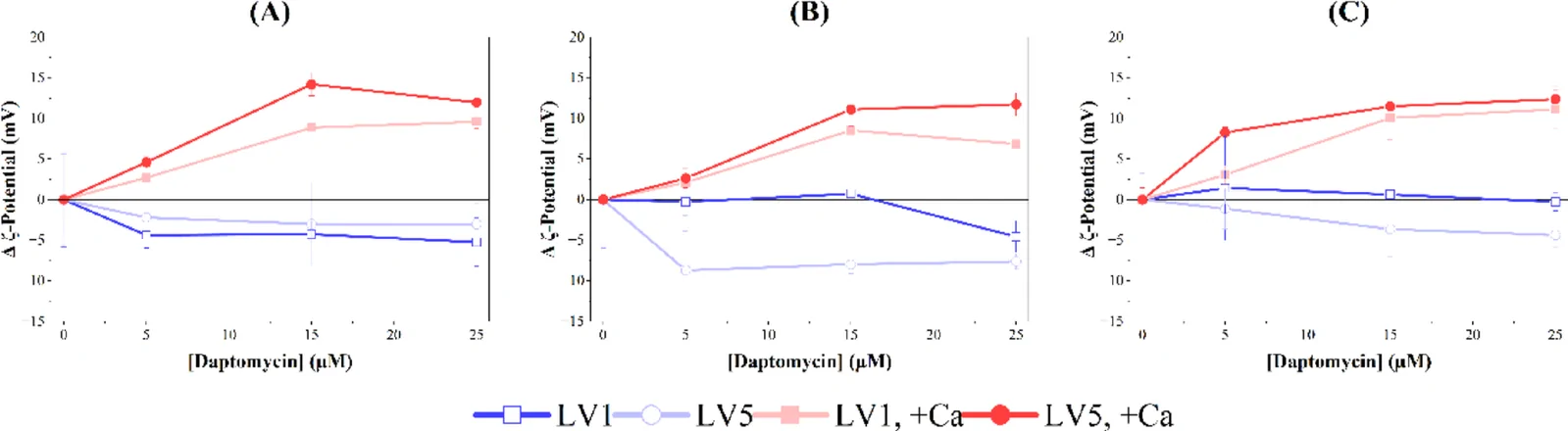
Δ ζ-Potential (mV) of 50 µM lipid vesicle #1 or #5 ± Ca^2+^ at **(A)** pH 5.0, **(B)** pH 7.0, and **(C)** pH 8.0 at various concentrations of daptomycin. Data are averages of 3 independent samples and error bars represent standard deviations

The interaction between daptomycin and the vesicles was also evaluated by measuring changes in the ζ (zeta)-potential of the vesicles. ζ-potential is the potential (mV) of the electrical double layer formed by the interaction of charged species with a charged surface (Dukhin and Xu 2025; Lyklema 1995). In this case, the vesicles had a negative ζ-potential arising from the PG lipids and soluble counterions associated with the surface forming the double layer. Changes in the ζ-potentials of lipid vesicles are shown in Fig. 4 for vesicles containing no cholesterol (lipid vesicle #1) and those containing 30% cholesterol (lipid vesicle #5), all measured at pH 5, 7, and 8. In all cases, the presence of Ca^2+^ ions induced a change in the ζ-potential of between + 10 and + 15 mV, indicating that the surface of the lipid vesicle became less negative as more daptomycin bound. In contrast, the changes in ζ-potential in the absence of calcium ranged from 0 to -10 mV, indicating weak interactions or interactions that made the surface slightly more negatively charged. It should be noted that the starting ζ-potentials in the absence of daptomycin differed in the presence and absence of calcium, likely reflecting interactions between some calcium ions and the PG headgroups on the vesicle surface, as shown in Supplemental Figure S4.

### Fluorescence Quenching

**Fig. 5:**
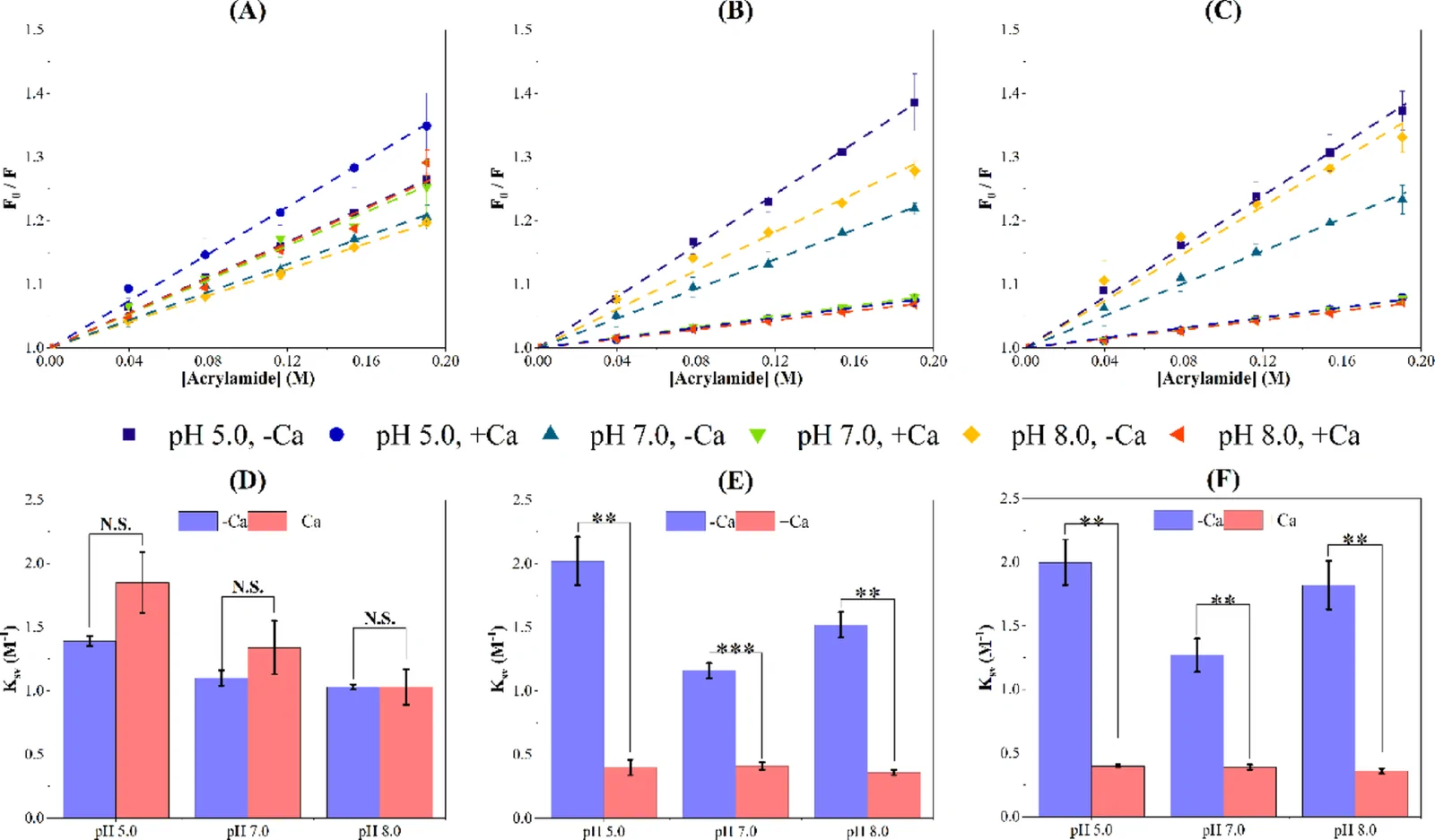
Fluorescence quenching of daptomycin (± 5.0 mM CaCl_2_) by acrylamide at pHs 5.0, 7.0, and 8.0. Stern-Volmer plots are shown in the (**A)** absence of lipid vesicles, **(B)** presence of lipid vesicle #1, and **(C)** presence of lipid vesicle #5. Stern-Volmer constants, *K*_*sv*_ (M^− 1^), are shown in the (**D)** absence of lipid vesicles, **(E)** presence of lipid vesicle #1, and **(F)** presence of lipid vesicle #5. Statistical significance determined by a two-tailed heteroscedastic T-test (NS if *p* > 0.05, * if *p* ≤ 0.05, ** if *p* ≤ 0.01, *** if *p* ≤ 0.001)

Although the binding interaction of daptomycin did not appear to be affected by solution pH or cholesterol content in the vesicle bilayers, a deeper understanding of how daptomycin interacts with the lipid bilayers was investigated using fluorescence quenching. Fluorescence quenching is a reduction in fluorescence emission intensity resulting from the fluorophore coming into close contact with a quencher (Lehrer 1971). First, daptomycin quenching by acrylamide, an aqueous quencher that does not effectively quench fluorophores embedded in the lipid bilayer, was evaluated. These data are shown in Fig. 5, with traditional Stern-Volmer plots in the top row and the K_sv_ (Stern-Volmer constant) data plotted in the bottom row. The first column of Fig. 5 (panels A & D) shows the results of quenching at pH 5, 7, and 8 in the absence of lipid vesicles, with and without 5mM CaCl_2_. In these samples, there is no bilayer to shield daptomycin from interactions with the acrylamide. This result demonstrated that the interaction between daptomycin and calcium did not affect acrylamide quenching. The middle column of Fig. 5 (panels B & E) shows quenching in the presence of lipid vesicles without cholesterol (lipid vesicle #1), while the final column of Fig. 5 (panels C & F) shows quenching in the presence of lipid vesicles with 30 mol% cholesterol. Consistent with the binding experiments in Fig. 3, the presence of cholesterol and the solution pH did not significantly affect acrylamide quenching. Notably, addition of CaCl_2_ resulted in a marked decrease in the K_sv_ values, consistent with daptomycin interacting with the bilayer surface and becoming more shielded from the aqueous acrylamide.

**Fig. 6:**
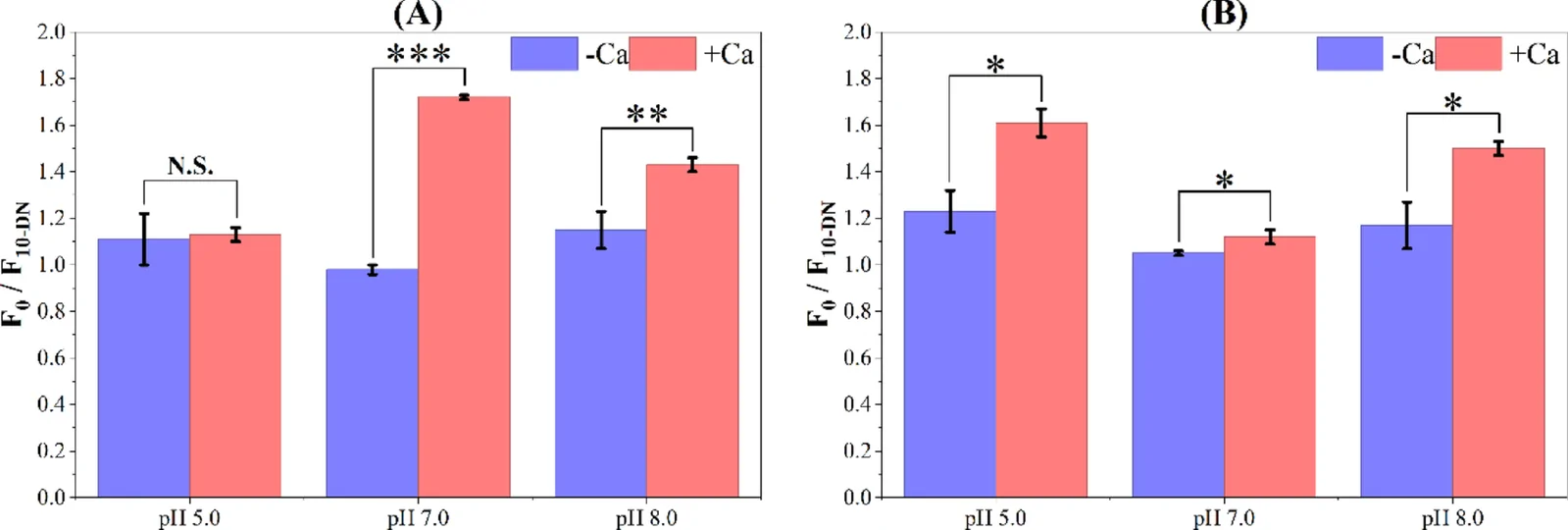
Fluorescence quenching of daptomycin (± 5.0 mM CaCl_2_) with 10-doxyl nonadecane (10-DN) at pHs 5.0, 7.0, and 8.0. Quenching quotient in the presence of **(A)** lipid vesicle #1 or **(B)** lipid vesicle #5. Statistical significance determined by a two-tailed heteroscedastic T-test (NS if *p* > 0.05, * if *p* ≤ 0.05, ** if *p* ≤ 0.01, *** if *p* ≤ 0.001)

Further evaluation of daptomycin orientation in the membrane was performed using the bilayer-embedded quencher 10-doxylnonadecane (10-DN). 10-DN has been shown to partition to the lipid bilayer interior and quenches molecules in contact with the hydrophobic core of the lipid bilayer(Caputo and London 2003). The data from the 10-DN quenching experiments are shown in Fig. 6. Here, an increase in the quenching quotient (*F*_*0*_*/F*_*10−DN*_) indicates the fluorophore is becoming more buried in the bilayer. In both lipid vesicle compositions tested (75:25 mol% POPC: POPG, Figs. 6A and 45:25:30 mol% POPC: POPG: Cholesterol, Fig. 6B), daptomycin becomes more exposed to the lipid bilayer core when interacting with Ca^2+^ ions. Consistent with prior results, the presence of cholesterol and changes in solution pH did not appear to have a consistent effect on the interaction of daptomycin with lipid bilayers. Although the quenching quotient did vary across individual pH values for lipid vesicles ± 30 mol% cholesterol. The 10-DN quenching data shown in Fig. 6 are consistent with the acrylamide quenching data in Fig. 5, indicating that when Ca^2+^ ions are present, the daptomycin fluorophores become more shielded from the aqueous environment and more exposed to interactions with the nonpolar core of the lipid bilayers.

### Circular Dichroism

**Fig. 7:**
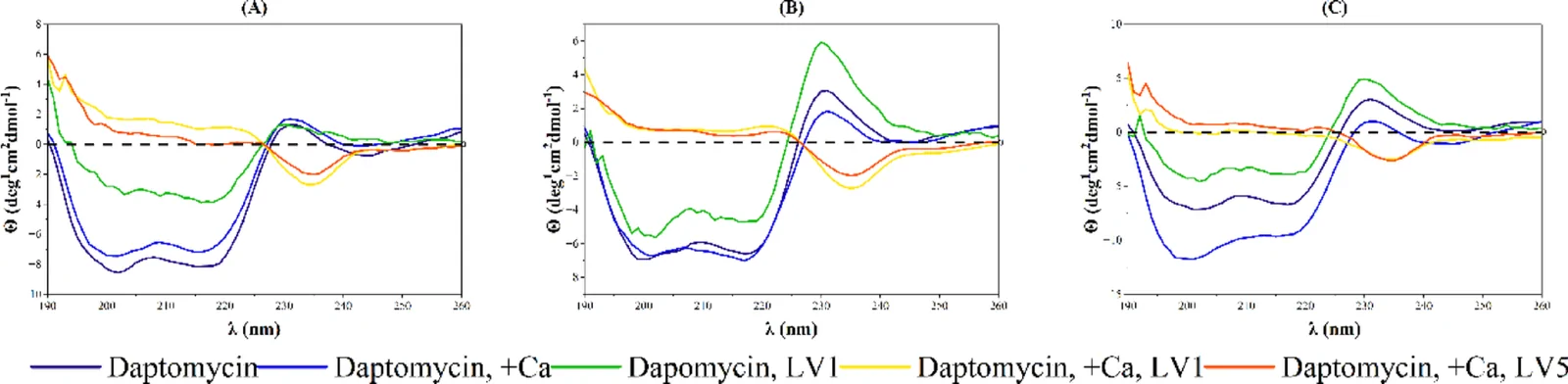
Circular dichroism spectra of daptomycin (purple), daptomycin + Ca^2+^ (blue), daptomycin + lipid vesicle #1 (green), daptomycin + Ca^2+^ + lipid vesicle #1 (yellow), and daptomycin + Ca^2+^ + lipid vesicle #5 (red) at **(A)** pH 5.0, **(B)** pH 7.0, or **(C)** pH 8.0

Circular dichroism (CD) spectroscopy has been extensively used to study AMPs; however, the cyclic backbone of daptomycin and the incorporation of D-amino acids into the structure result in “atypical” CD spectra. However, there are still stark differences between the CD spectra of daptomycin in solution compared to when daptomycin is bound to the lipid bilayer surface, providing utility when used in conjunction with the binding studies. CD spectra were additionally collected on the daptomycin peptide in solution, bound to lipid bilayers, and ± Ca^2+^ ions, as shown in Fig. 7. In solution, the CD spectrum of daptomycin exhibits two minima at 201 nm and 215 nm as well as a maximum at 231 nm, regardless of the presence of calcium. The shape of the CD spectra of daptomycin changes significantly upon binding to lipid vesicles, resulting in a minimum at ~ 235 nm. This spectral transition was observed across all tested pH values and was unaffected by lipid bilayer composition.

### Membrane Permeabilization

**Fig. 8:**
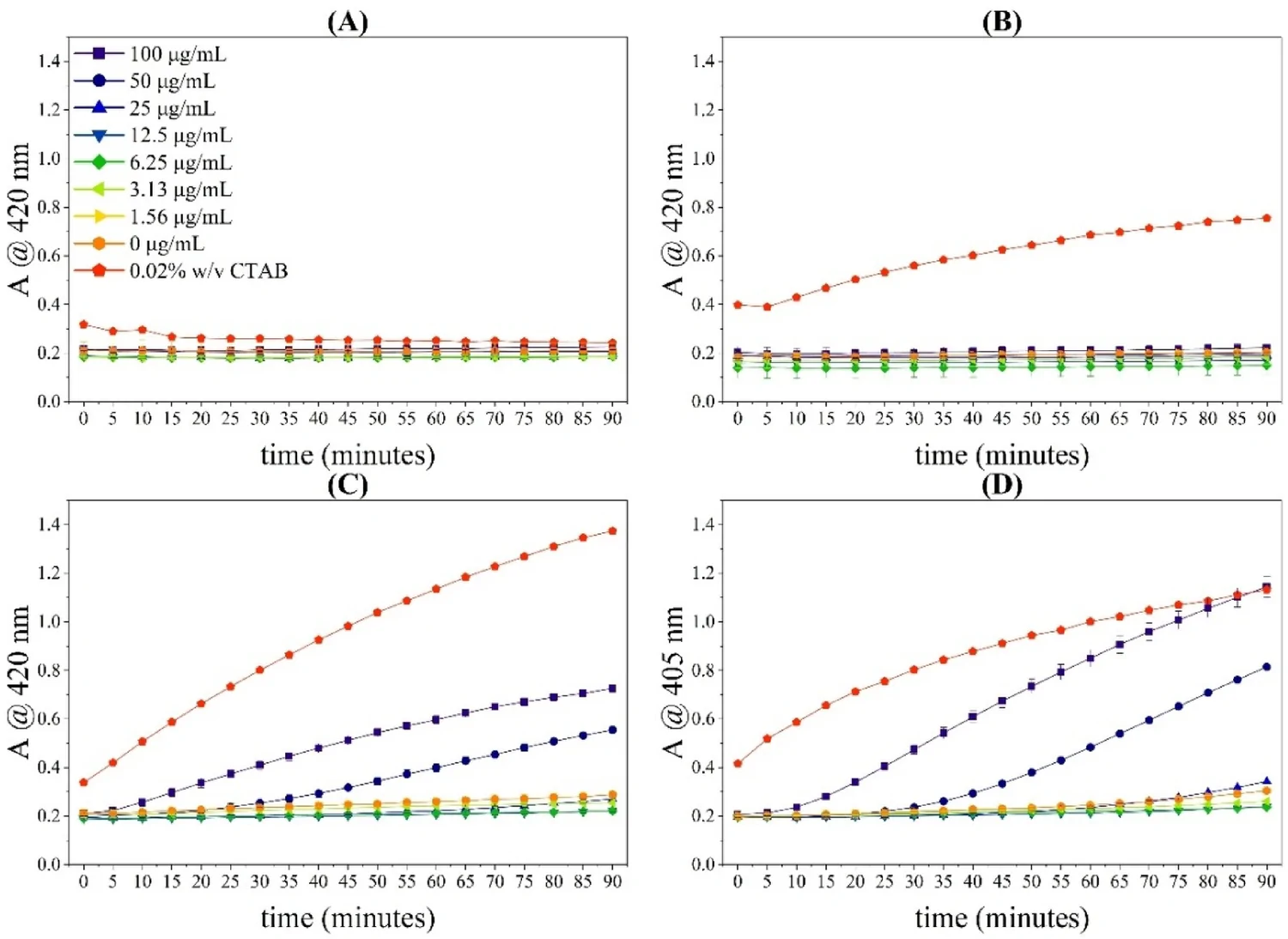
Permeabilization of the bacterial membrane of *Staphylococcus aureus* AS22-1 CaM by various concentrations of daptomycin, observed through the conversion of the substrate, ONPG, to the chromogenic product, ONP, by cytosolic enzyme β-galactosidase at **(A)** pH 4.0, **(B)** pH 5.0, **(C)** pH 7.0, and **(D)** pH 8.0

The ability of daptomycin to permeabilize bacterial membranes at different pH conditions was also investigated. Here, we used a genetically modified strain of *S. aureus* which was engineered to produce the cytoplasmic enzyme β-galactosidase (O’Neill et al. 2004). Using the chromogenic substrate ONPG, which has poor spontaneous permeability across the *S. aureus* membrane, daptomycin-induced permeabilization can be evaluated by observing the change in the conversion rate of substrate (ONPG) into the chromophore product (ONP). The results of these experiments are shown in Fig. 8. At pH values 7 and 8, daptomycin clearly induces membrane destabilization/disruption in a dose-dependent manner. However, at pH 5, daptomycin did not induce any detectable permeabilization of the *S. aureus* membrane compared to control samples. While experiments at pH 4 were carried out, the positive control (CTAB) indicates that β-galactosidase was likely denatured or otherwise inactivated at this pH, which is consistent with prior findings, making the analysis impossible using this method at this lowest pH (Tenu et al. 1971). The results of these experiments were confirmed using the DNA-binding dye propidium iodide, as shown in supplemental figure S5.

## Discussion

While daptomycin has been a clinically approved antibiotic for several decades, there is still a great deal of fundamental and biophysical understanding of this molecule that remains unexplored. Some of the challenges in studying daptomycin include the relative lack of flexibility in performing structure-activity relationships due to the cyclic nature of the peptide as well as the incorporation of numerous non-canonical amino acids. The limited SAR studies that have been performed show significant sensitivity at many positions within the daptomycin macrocycle, resulting in very few next-generation analogs developed from the parent molecule (Chow et al. 2020a, b; Karas et al. 2020; Lin et al. 2017).

Understanding the interactions of daptomycin with lipid bilayers initially focused on the strict dependence on phosphatidylglycerol lipids in the target membrane and on the importance of Ca^2+^ ions on the binding and activity of daptomycin. However, few biophysical studies have been conducted to understand this interaction and other factors that influence daptomycin-bilayer interactions. It was recently demonstrated by Kotsogianni and coworkers, using isothermal titration calorimetry, that calcium does not interact with daptomycin in the absence of POPG-containing lipid bilayers, thereby effectively requiring a tripartite interaction between daptomycin, Ca^2+^ ions, and PG headgroups (Kotsogianni et al. 2021). Our results presented in this work support this model in which daptomycin does not interact with lipid bilayers containing PG headgroups in the absence of Ca^2+^ ions (Fig. 3), and that Ca^2+^ ions alone are insufficient to induce any structural changes in daptomycin that are observable by fluorescence or CD spectroscopy (Figs. 2 and 7).

While lipids containing PG-headgroups are clearly essential for daptomycin binding and membrane interaction, natural lipid membranes are complex mixtures of proteins, lipids, and sterols. Sterol incorporation into bilayers has been shown to yield longer pores for some peptides (Zan et al. 2025), while inhibiting pore formation in others, such as melittin and magainin (Henderson et al. 2019; Li et al. 2021; McHenry et al. 2012). Similarly, membrane fluidity affects the pore-forming mechanism of toxins and the binding of small, cyclic peptides (Beck et al. 2025; Pedrera et al. 2023). With respect to binding interactions, multiple peptide systems demonstrate modulated binding affinity to cholesterol-containing membranes, such as gp41, ApoE, and aβ (Lockhart and Klimov 2017; Meher et al. 2019; Mirdha et al. 2022). This is also true of many pore-forming peptides, with reduced binding affinity for cholesterol-containing bilayers/membranes complicating pore-formation studies (Arsov et al. 2008; Sharma et al. 2015). In these systems, peptide interactions with the bilayer involve the α-helical backbone and multiple side chains inserting into the bilayer. In the case of daptomycin presented here, the binding and membrane insertion of the peptide is driven in part by the N-terminal C10 chain on the molecule (Fig. 1). As the data demonstrates little effect of cholesterol on binding affinity (Fig. 3), this is consistent with a model in which the alkyl chain of daptomycin easily inserts into the membrane regardless of lipid packing or membrane fluidity. These data are consistent with the model in which daptomycin shallowly inserts into the membrane (Beriashvili et al. 2020; Huang 2020). Overall, the lack of dependence on cholesterol, and subsequently the differences in membrane fluidity, on daptomycin binding indicates a robust molecular evolution that maintains the natural activity of daptomycin under a wide range of environmental conditions.

The influence of pH on daptomycin binding and activity is also a complex phenomenon. The relationship between the solution aggregation state of daptomycin and its binding to lipid bilayers has been the subject of numerous studies. As mentioned above, ITC studies have shown that daptomycin does not interact with calcium in solution; however, the exact binding stoichiometry of daptomycin, Ca^2+^ ions, and PG headgroups remains under investigation, with several stoichiometric ratios reported in the literature (Ball et al. 2004; Kotsogianni et al. 2021; Lee et al. 2017; Moreira and Taylor 2022). The binding of daptomycin to calcium is a chelation interaction involving the carboxylate-containing residues Asp3, Asp7, Asp9 and mGlu12, which is inherently dependent on pH, which affects the protonation state of the carboxylates. The pK_a_ values of the daptomycin carboxyl amino acids were determined to be ~ 1.0 through 4.8 using ^1^H NMR pH titration by Qui and peers in 2011(Qiu et al. 2011). Our results showed almost no variation in apparent affinity for bilayers between pH 5 and pH 8, indicating that solution pH does not affect daptomycin’s binding affinity for lipid bilayers and supporting the model in which Ca^2+^ ions do not interact with the peptide in solution. These binding constants are similar, albeit somewhat lower, than those reported by Zhang and coworkers, likely due to differences in lipid composition and Ca^2+^ ion concentration between their study and this one (Zhang et al. 2017). The ζ-potential measurements confirmed that Ca^2+^ ions still bound to the lipid vesicles under all pH conditions, as evidenced by the change in initial potential in the presence and absence of calcium (Fig. 4).

Once bound to the bilayer, the mechanism of daptomycin is still not fully understood. Molecular dynamics (MD) simulations have shown that daptomycin inserts into the bilayer via the C10 tail and is stabilized in this conformation by the formation of a daptomycin-PG headgroup complex, yielding a model in which Trp3 is shallowly embedded in the bilayer(Liu and Karttunen 2018). The fluorescence quenching data in Figs. 5 and 6 support the model in which Trp3 localizes to the headgroup region/bilayer interface. Specifically, the acrylamide quenching data show a dramatic shift in quencher accessibility, as evidenced by a decrease in K_sv_ upon daptomycin interaction with lipid bilayers. Similarly, 10-DN fluorescence quenching supports this model, with quenching values similar to those observed in peptides with shallowly inserted Trp residues (Caputo and London 2004; Hitchner et al. 2019). Interestingly, the largest and most significant calcium-induced changes in 10-DN quenching were observed at pH 7 in bilayers lacking cholesterol. This could indicate the presence of cholesterol mildly inhibits daptomycin insertion, although the trend is not evident at other pH values tested. The 10-DN fluorescence quenching results also support the MD simulation models in the literature. In the MD model, daptomycin adopts a shallower conformation in the lipid bilayers in the absence of Ca^2+^ ions, which is supported by the increased 10-DN quenching in the absence of Ca^2+^ ions (Liu and Karttunen 2018). Taken together, the quenching results support a model in which daptomycin inserts into the lipid bilayer primarily through the C10 tail and has an interfacial orientation of the peptide macrocycle.

The role of bacterial membrane permeabilization in the mechanism of daptomycin has been a significant area of interest and investigation. While many amphiphilic α-helical AMPs have been shown to exert antibacterial activity through membrane disruption, daptomycin is inherently different due to the peptide macrocycle in the molecule. Early work on the mechanism demonstrated disruption of bacterial membranes as judged by DNA-binding fluorophores, but these studies required significant incubation period (Ledger and Edwards 2023). Alternatively, several studies employed membrane potential-sensitive probes such as DiSC_3_(5) (3,3’-Dipropylthiadicarbocyanine Iodide), but these potential-sensitive probes are highly sensitive to ion flux and cannot discriminate between small membrane perturbations and larger sized pores(Ledger and Edwards 2023; Silverman et al. 2003). The results of the *S. aureus* membrane permeabilization experiments presented support the model in which daptomycin forms discrete pores in the bacterial membrane. This pore formation is dose-dependent and consistent with models in which daptomycin forms small oligomers on the membrane surface (Ho et al. 2008; Nakamuro et al. 2022; Zhang et al. 2017). Additionally, pore formation in *S. aureus* membranes is rapid, occurring on the same timescale as the detergent controls (Fig. 8). While clearly demonstrating that daptomycin forms stable pores in Gram-positive bacterial membranes, this does not exclude the possibility of additional mechanisms of action (Buttress et al. 2025).

The results of the work presented here should also be interpreted in the context of the more recent sets of studies which indicate the formation of a tripartite complex between Ca^2+^-daptomycin, PG lipids, and undecaprenyl-coupled intermediates of peptidoglycan synthesis. In the aim of clarity, the term “tripartite complex” can also be interpreted as a quaternary complex if the daptomycin and Ca^2+^ ions are considered independently. Several studies have shown that when daptomycin forms a complex with PG lipids and undecaprenyl-coupled intermediates, there are downstream effects on cell wall biosynthesis including reduced crosslinking in the cell wall as well as accumulation at and disruption of the division septum during bacterial cell division (Buttress et al. 2025). While the exact molecular details of the interaction is still unknown, undecaprenyl phosphate and related molecules have been shown to increase membrane fluidity and ion permeability, although those studies primarily focused on dolichol and dolichyl phosphate which have 95 C in the isoprenoid tail compared to 55 C in the tails of undecaprenyl phosphate (Valtersson et al. 1985; Wang et al. 2008). Nevertheless, maintenance of appropriate membrane fluidity has been indicated as an important factor in bacterial cell division through lipid- and protein mediated mechanisms (Gohrbandt et al. 2022; Zielinska et al. 2020). This would imply that daptomycin can function under highly dynamic membrane conditions. The results presented in this study indicate that increases in cholesterol and the resulting decreases in bilayer fluidity would not impact daptomycin binding and insertion into bilayers, although the impact on formation of the tripartite complex was not investigated. Additionally, environmental pH has also been demonstrated to affect bacterial cell division and has recently been indicated to influence FtsZ accumulation at the cell division septum, the same location where the daptomycin tripartite complex is believed to function (Mueller et al. 2019, 2020). Taken together, the robustness of daptomycin membrane interactions despite pH or sterol and fluidity changes in the bilayer are consistent with the tripartite complex model and the associated membrane and environmental effects at the bacterial cell division site.

The data presented highlights several important insights into the mechanism and membrane interactions of daptomycin. The studies presented demonstrate that daptomycin can effectively bind to lipid bilayers under a variety of conditions, both environmental and with respect to the bilayer composition. Importantly, the insensitivity to pH and membrane sterol composition is likely valuable to the natural function of daptomycin as a means of offensive action by *S. roseosporus* against other Gram-positive strains in the local environment. This insensitivity can help ensure that daptomycin effectively provides *S. roseosporus* with a competitive advantage in nature which is proposed to have evolved/diverged ~1B years ago (Baltz 2010). Additionally, the demonstration of Trp depth changes in the bilayer in response to Ca^2+^ ion concentrations supports the proposed models in which the daptomycin oligomer becomes more deeply inserted into the bilayer upon Ca^2+^ interaction, thereby promoting pore formation. While these studies show that daptomycin can bind to membranes under a wide range of conditions, there are still numerous open questions regarding the mechanism of action of daptomycin including the importance of daptomycin-mediated pore formation, the molecular interactions between daptomycin and undecaprenyl phosphate, and a more detailed understanding of the daptomycin oligomer/aggregate at the membrane surface.

## Supplementary Information

Below is the link to the electronic supplementary material.


Supplementary Material 1



Supplementary Material 2


## Data Availability

Data supporting the findings of this study are available within the paper and its Supplementary Information, or available upon request from the authors.

## References

[CR1] Al Musaimi O (2025) FDA-Approved Antibacterials and Echinocandins. Antibiotics (Basel), p 14 10.3390/antibiotics14020166 PMC11851826 40001410

[CR2] Alaoui Mdarhri H, Benmessaoud R, Yacoubi H, Seffar L, Guennouni Assimi H, Hamam M, Boussettine R, Filali-Ansari N, Lahlou FA, Diawara I, Ennaji MM, Kettani-Halabi M (2022) Alternatives Therapeutic Approaches to Conventional Antibiotics: Advantages, Limitations and Potential Application in Medicine. Antibiotics (Basel) 11 10.3390/antibiotics11121826 PMC9774722 36551487

[CR3] Antimicrobial Resistance C (2022) Global burden of bacterial antimicrobial resistance in 2019: a systematic analysis. Lancet 399:629–655 35065702 10.1016/S0140-6736(21)02724-0 PMC8841637

[CR4] Arsov Z, Nemec M, Schara M, Johansson H, Langel U, Zorko M (2008) Cholesterol prevents interaction of the cell-penetrating peptide transportan with model lipid membranes. J Pept Sci 14:1303–1308 18683276 10.1002/psc.1062

[CR5] Ball LJ, Goult CM, Donarski JA, Micklefield J, Ramesh V (2004) NMR structure determination and calcium binding effects of lipopeptide antibiotic daptomycin. Org Biomol Chem 2:1872–1878 15227539 10.1039/b402722a

[CR6] Baltz RH (2010) Genomics and the ancient origins of the daptomycin biosynthetic gene cluster. J Antibiot (Tokyo) 63:506–511 20648020 10.1038/ja.2010.82

[CR7] Beck K, Nandy J, Hoernke M (2025) Strong Membrane Permeabilization Activity Can Reduce Selectivity of Cyclic Antimicrobial Peptides. J Phys Chem B 129:2446–2460 39969852 10.1021/acs.jpcb.4c05019 PMC11891913

[CR8] Beriashvili D, Spencer NR, Dieckmann T, Overduin M, Palmer M (2020) Characterization of multimeric daptomycin bound to lipid nanodiscs formed by calcium-tolerant styrene-maleic acid copolymer. Biochim Biophys Acta Biomembr 1862:183234 32145282 10.1016/j.bbamem.2020.183234

[CR9] Bogdanov M, Pyrshev K, Yesylevskyy S, Ryabichko S, Boiko V, Ivanchenko P, Kiyamova R, Guan Z, Ramseyer C, Dowhan W (2020) Phospholipid distribution in the cytoplasmic membrane of Gram-negative bacteria is highly asymmetric, dynamic, and cell shape-dependent. Sci Adv 6:eaaz6333 32537497 10.1126/sciadv.aaz6333 PMC7269648

[CR10] Buttress JA, Schafer AB, Koh A, Wheatley J, Mickiewicz K, Wenzel M, Strahl H (2025) The last resort antibiotic daptomycin exhibits two independent antibacterial mechanisms of action. Nat Commun 16:10320 41285733 10.1038/s41467-025-65287-w PMC12645038

[CR11] Caputo GA, London E (2003) Using a novel dual fluorescence quenching assay for measurement of tryptophan depth within lipid bilayers to determine hydrophobic alpha-helix locations within membranes. Biochemistry 42:3265–3274 12641458 10.1021/bi026696l

[CR12] Caputo GA, London E (2004) Position and ionization state of Asp in the core of membrane-inserted alpha helices control both the equilibrium between transmembrane and nontransmembrane helix topography and transmembrane helix positioning. Biochemistry 43:8794–8806 15236588 10.1021/bi049696p

[CR13] Caputo GA, London E (2019) Analyzing Transmembrane Protein and Hydrophobic Helix Topography by Dual Fluorescence Quenching. Methods Mol Biol 2003:351–368 31218625 10.1007/978-1-4939-9512-7_15

[CR14] Chow HY, Po KHL, Gao P, Blasco P, Wang X, Li C, Ye L, Jin K, Chen K, Chan EWC, You X, Kao YT, Chen R, Li S, X (2020a) Methylation of Daptomycin Leading to the Discovery of Kynomycin, a Cyclic Lipodepsipeptide Active against Resistant Pathogens. J Med Chem 63:3161–3171 32097000 10.1021/acs.jmedchem.9b01957

[CR15] Chow HY, Po KHL, Jin K, Qiao G, Sun Z, Ma W, Ye X, Zhou N, Chen S, Li X (2020b) Establishing the Structure-Activity Relationship of Daptomycin. ACS Med Chem Lett 11:1442–1449 32676152 10.1021/acsmedchemlett.0c00175 PMC7357220

[CR16] Dukhin AS, Xu R (2025) Chapter 1 - Introduction to electrokinetics. In: Dukhin AS, Xu R (eds) Interface Science and Technology. Elsevier, pp 3–8

[CR17] Eisenstein BI, Oleson FB Jr., Baltz RH (2010) Daptomycin: from the mountain to the clinic, with essential help from Francis Tally, MD. Clin Infect Dis 50(Suppl 1):S10–S15 20067387 10.1086/647938

[CR18] Ghosh C, Sarkar P, Issa R, Haldar J (2019) Alternatives to Conventional Antibiotics in the Era of Antimicrobial Resistance. Trends Microbiol 27:323–338 30683453 10.1016/j.tim.2018.12.010

[CR19] Gohrbandt M, Lipski A, Grimshaw JW, Buttress JA, Baig Z, Herkenhoff B, Walter S, Kurre R, Deckers-Hebestreit G, Strahl H (2022) Low membrane fluidity triggers lipid phase separation and protein segregation in living bacteria. EMBO J 41:e109800 35037270 10.15252/embj.2021109800 PMC8886542

[CR20] Henderson JM, Iyengar NS, Lam KLH, Maldonado E, Suwatthee T, Roy I, Waring AJ, Lee KYC (2019) Beyond electrostatics: Antimicrobial peptide selectivity and the influence of cholesterol-mediated fluidity and lipid chain length on protegrin-1 activity. Biochim Biophys Acta Biomembr 1861:182977 31077677 10.1016/j.bbamem.2019.04.011

[CR22] Hitchner MA, Santiago-Ortiz LE, Necelis MR, Shirley DJ, Palmer TJ, Tarnawsky KE, Vaden TD, Caputo GA (2019) Activity and characterization of a pH-sensitive antimicrobial peptide. Biochim Biophys Acta Biomembr 1861:182984 31075228 10.1016/j.bbamem.2019.05.006 PMC6721999

[CR21] Hitchner MA, Necelis MR, Shirley D, Caputo GA (2021) Effect of Non-natural Hydrophobic Amino Acids on the Efficacy and Properties of the Antimicrobial Peptide C18G. Probiotics Antimicrob Proteins 13:527–541 32889698 10.1007/s12602-020-09701-3 PMC7933317

[CR24] Ho SW, Jung D, Calhoun JR, Lear JD, Okon M, Scott WR, Hancock RE, Straus SK (2008) Effect of divalent cations on the structure of the antibiotic daptomycin. Eur Biophys J 37:421–433 17968536 10.1007/s00249-007-0227-2

[CR23] Ho CS, Wong CTH, Aung TT, Lakshminarayanan R, Mehta JS, Rauz S, McNally A, Kintses B, Peacock SJ, de la Fuente-Nunez C, Hancock REW, Ting DSJ (2025) Antimicrobial resistance: a concise update. Lancet Microbe 6:100947 39305919 10.1016/j.lanmic.2024.07.010

[CR25] Huang HW (2020) DAPTOMYCIN, its membrane-active mechanism vs. that of other antimicrobial peptides. Biochim Biophys Acta Biomembr 1862:183395 32526177 10.1016/j.bbamem.2020.183395

[CR26] Kaderabkova N, Mahmood AJS, Mavridou DAI (2024) Antibiotic susceptibility testing using minimum inhibitory concentration (MIC) assays. NPJ Antimicrob Resist 2:37 39843555 10.1038/s44259-024-00051-6 PMC11721449

[CR27] Karas JA, Carter GP, Howden BP, Turner AM, Paulin OKA, Swarbrick JD, Baker MA, Li J, Velkov T (2020) Structure-Activity Relationships of Daptomycin Lipopeptides. J Med Chem 63:13266–13290 32687352 10.1021/acs.jmedchem.0c00780

[CR28] Kotsogianni I, Wood TM, Alexander FM, Cochrane SA, Martin NI (2021) Binding Studies Reveal Phospholipid Specificity and Its Role in the Calcium-Dependent Mechanism of Action of Daptomycin. ACS Infect Dis 7:2612–2619 34406007 10.1021/acsinfecdis.1c00316 PMC8438661

[CR29] Kuroda K, Caputo GA (2013) Antimicrobial polymers as synthetic mimics of host-defense peptides. Wiley Interdiscip Rev Nanomed Nanobiotechnol 5:49–66 23076870 10.1002/wnan.1199

[CR30] Lai CC, Chen SY, Ko WC, Hsueh PR (2021) Increased antimicrobial resistance during the COVID-19 pandemic. Int J Antimicrob Agents 57:106324 33746045 10.1016/j.ijantimicag.2021.106324 PMC7972869

[CR31] Langford BJ, So M, Simeonova M, Leung V, Lo J, Kan T, Raybardhan S, Sapin ME, Mponponsuo K, Farrell A, Leung E, Soucy JR, Cassini A, MacFadden D, Daneman N, Bertagnolio S (2023) Antimicrobial resistance in patients with COVID-19: a systematic review and meta-analysis. Lancet Microbe 4:e179–e191 36736332 10.1016/S2666-5247(22)00355-X PMC9889096

[CR32] Latour RA (2015) The Langmuir isotherm: a commonly applied but misleading approach for the analysis of protein adsorption behavior. J Biomed Mater Res A 103:949–958 24853075 10.1002/jbm.a.35235

[CR33] Ledger EVK, Edwards AM (2023) Growth Arrest of Staphylococcus aureus Induces Daptomycin Tolerance via Cell Wall Remodelling. mBio 14:e0355822 36722949 10.1128/mbio.03558-22 PMC9973334

[CR34] Lee MT, Hung WC, Hsieh MH, Chen H, Chang YY, Huang HW (2017) Molecular State of the Membrane-Active Antibiotic Daptomycin. Biophys J 113:82–90 28700928 10.1016/j.bpj.2017.05.025 PMC5510707

[CR35] Lehrer SS (1971) Solute perturbation of protein fluorescence. The quenching of the tryptophyl fluorescence of model compounds and of lysozyme by iodide ion. Biochemistry 10:3254–3263 5119250 10.1021/bi00793a015

[CR36] Li J, Lu X, Ma W, Chen Z, Sun S, Wang Q, Yuan B, Yang K (2021) Cholesterols Work as a Molecular Regulator of the Antimicrobial Peptide-Membrane Interactions. Front Mol Biosci 8:638988 33634166 10.3389/fmolb.2021.638988 PMC7902056

[CR37] Lin D, Lam HY, Han W, Cotroneo N, Pandya BA, Li X (2017) Structure-activity relationship of daptomycin analogues with substitution at (2S, 3R) 3-methyl glutamic acid position. Bioorg Med Chem Lett 27:456–459 28038833 10.1016/j.bmcl.2016.12.046

[CR38] Liu B, Karttunen M (2018) Lipopeptide daptomycin: Interactions with bacterial and phospholipid membranes, stability of membrane aggregates and micellation in solution. Biochim Biophys Acta Biomembr 1860:1949–1954 29627322 10.1016/j.bbamem.2018.03.028

[CR39] Lockhart C, Klimov DK (2017) Cholesterol Changes the Mechanisms of Abeta Peptide Binding to the DMPC Bilayer. J Chem Inf Model 57:2554–2565 28910085 10.1021/acs.jcim.7b00431

[CR40] Lv K, Fang Y, He C, Xin J, Xue C, Dong N (2026) Unlocking the potential of antimicrobial lipopeptides: a pathway to combat multidrug-resistant pathogens. Biochem Pharmacol 248:117849 41763368 10.1016/j.bcp.2026.117849

[CR41] Lyklema J (1995) 3 - Electric Double Layers. In: J Lyklema (eds) Fundamentals of Interface and Colloid Science, Academic Press, 3-1-3-232

[CR42] MacLean RC, San Millan A (2019) The evolution of antibiotic resistance. Science 365:1082–1083 31515374 10.1126/science.aax3879

[CR43] McHenry AJ, Sciacca MF, Brender JR, Ramamoorthy A (2012) Does cholesterol suppress the antimicrobial peptide induced disruption of lipid raft containing membranes? Biochim Biophys Acta 1818:3019–3024 22885355 10.1016/j.bbamem.2012.07.021 PMC3455134

[CR44] Meher G, Sinha S, Pattnaik GP, Ghosh Dastidar S, Chakraborty H (2019) Cholesterol Modulates Membrane Properties and the Interaction of gp41 Fusion Peptide To Promote Membrane Fusion. J Phys Chem B 123:7113–7122 31345037 10.1021/acs.jpcb.9b04577

[CR45] Mirdha L, Sengupta T, Chakraborty H (2022) Lipid composition dependent binding of apolipoprotein E signal peptide: Importance of membrane cholesterol in protein trafficking. Biophys Chem 291:106907 36228459 10.1016/j.bpc.2022.106907

[CR46] Moreira R, Taylor SD (2022) Establishing the Structure-Activity Relationship between Phosphatidylglycerol and Daptomycin. ACS Infect Dis 8:1674–1686 35793519 10.1021/acsinfecdis.2c00262

[CR47] Mueller EA, Westfall CS, Levin PA (2019) Environmental pH impacts division assembly and cell size in Escherichia coli. bioRxiv:747808

[CR48] Mueller EA, Westfall CS, Levin PA (2020) pH-dependent activation of cytokinesis modulates Escherichia coli cell size. PLoS Genet 16:e1008685 32203516 10.1371/journal.pgen.1008685 PMC7117782

[CR49] Nakamuro T, Kamei K, Sun K, Bode JW, Harano K, Nakamura E (2022) Time-Resolved Atomistic Imaging and Statistical Analysis of Daptomycin Oligomers with and without Calcium Ions. J Am Chem Soc 144:13612–13622 35857028 10.1021/jacs.2c03949

[CR50] O’Neill AJ, Miller K, Oliva B, Chopra I (2004) Comparison of assays for detection of agents causing membrane damage in Staphylococcus aureus. J Antimicrob Chemother 54:1127–1129 15531595 10.1093/jac/dkh476

[CR51] Pedrera L, Ros U, Fanani ML, Lanio ME, Epand RM, Garcia-Saez AJ, Alvarez C (2023) The Important Role of Membrane Fluidity on the Lytic Mechanism of the alpha-Pore-Forming Toxin Sticholysin I. Toxins (Basel) 15 10.3390/toxins15010080 PMC9865829 36668899

[CR52] Qiu J, Yu L, Kirsch LE (2011) Estimated pKa values for specific amino acid residues in daptomycin. J Pharm Sci 100:4225–4233 21547914 10.1002/jps.22608

[CR53] Richardson LA (2017) Understanding and overcoming antibiotic resistance. PLoS Biol 15:e2003775 28832581 10.1371/journal.pbio.2003775 PMC5584968

[CR54] Salam MA, Al-Amin MY, Salam MT, Pawar JS, Akhter N, Rabaan AA, Alqumber MAA (2023) Antimicrobial Resistance: A Growing Serious Threat for Global Public Health. Healthcare (Basel) 11 10.3390/healthcare11131946 PMC10340576 37444780

[CR55] Sharma VK, Mamontov E, Anunciado DB, O’Neill H, Urban VS (2015) Effect of antimicrobial peptide on the dynamics of phosphocholine membrane: role of cholesterol and physical state of bilayer. Soft Matter 11:6755–6767 26212615 10.1039/c5sm01562f

[CR56] Silverman JA, Perlmutter NG, Shapiro HM (2003) Correlation of daptomycin bactericidal activity and membrane depolarization in Staphylococcus aureus. Antimicrob Agents Chemother 47:2538–2544 12878516 10.1128/AAC.47.8.2538-2544.2003 PMC166110

[CR57] Smith RA, M’Ikanatha N, Read M, AF (2015) Antibiotic resistance: a primer and call to action. Health Commun 30:309–314 25121990 10.1080/10410236.2014.943634 PMC4275377

[CR58] Tenu JP, Viratelle OM, Garnier J, Yon J (1971) pH dependence of the activity of beta-galactosidase from Escherichia coli. Eur J Biochem 20:363–370 4931951 10.1111/j.1432-1033.1971.tb01402.x

[CR59] Valtersson C, van Duyn G, Verkleij AJ, Chojnacki T, de Kruijff B, Dallner G (1985) The influence of dolichol, dolichol esters, and dolichyl phosphate on phospholipid polymorphism and fluidity in model membranes. J Biol Chem 260:2742–2751 3919007

[CR60] Wang X, Mansourian AR, Quinn PJ (2008) The effect of dolichol on the structure and phase behaviour of phospholipid model membranes. Mol Membr Biol 25:547–556 18985511 10.1080/09687680802520684

[CR61] Zan B, Ulmschneider MB, Ulmschneider JP (2025) The difference between MelP5 and melittin membrane poration. Sci Rep 15:7442 40033017 10.1038/s41598-025-91951-8 PMC11876596

[CR62] Zhang J, Scott WRP, Gabel F, Wu M, Desmond R, Bae J, Zaccai G, Algar WR, Straus SK (2017) On the quest for the elusive mechanism of action of daptomycin: Binding, fusion, and oligomerization. Biochim Biophys Acta Proteins Proteom 1865:1490–1499 28844744 10.1016/j.bbapap.2017.07.020

[CR63] Zielinska A, Savietto A, de Sousa Borges A, Martinez D, Berbon M, Roelofsen JR, Hartman AM, de Boer R, Van der Klei IJ, Hirsch AK, Habenstein B, Bramkamp M, Scheffers DJ (2020) Flotillin-mediated membrane fluidity controls peptidoglycan synthesis and MreB movement. Elife 9 10.7554/eLife.57179 PMC7360373 32662773

