# Supplementary material for: Effects of Cholesterol and Solution pH on Daptomycin-bilayer Interactions
Source: J Membr Biol. 2026 Aug 29;259(1):34. doi: 10.1007/s00232-026-00400-8 (PMC13526048; doi:10.1007/s00232-026-00400-8)
Supplement: Supplementary file 2 [file 232_2026_400_MOESM2_ESM.docx]

Effects of cholesterol and solution pH on daptomycin-bilayer interactions.

Samuel R. Pennock†^1^, Maggie Cortes†^1^, Megha D. Salecha†^1^, Neil X. Sevilla^2^, Nicole R. Rementer^1^, Kevin Rana^2^, Luke C. Hicks^2^, Emmaleigh Fox^2^, Ahmed Grine^1^, Patience S. Hammel^1^, Timothy D. Vaden^1^, Benjamin R. Carone^2^, and Gregory A. Caputo^1,2^*

1. Department of Chemistry & Biochemistry, Rowan University Glassboro NJ USA
2. Department of Biological & Biomedical Science, Rowan University Glassboro NJ USA

Figure S1

Figure S1:Representative spectra of daptomycin under various conditions. Normalized spectra of daptomycin ± lipid vesicles (45% POPC: 25% POPG: 30% Cholesterol) in buffer (20 mM Tris, pH 7.0) with **(A)** 150 mM NaCl and 0.0 mM CaCl_2_ or **(B)** 140 mM NaCl and 5.0 mM CaCl_2_.

Figure S2

Figure S2: CPS (counts per second) and normalized intensity of daptomycin in either buffer 5 (20 mM Tris, 150 mM NaCl, pH 7.0) or buffer 6 (20 mM Tris, 140 mM NaCl, 5 mM CaCl_2_, pH 7.0) with set concentrations of lipid vesicle #1 (75% POPC: 25% POPG) titrated into solution. λ_ex._ = 280nm. **(A)** CPS in buffer 5, **(B)** normalized intensity in buffer 5, **(C)** CPS in buffer 6, and **(D)** normalized intensity in buffer 6.

Figure S3

Figure S3. The fraction of daptomycin bound to lipid vesicles 1 through 5 at **(A)** pH 5.0, **(B)** pH 7.0, and **(C)** 8.0. Data fitting was Data points represent averages of at least 3 independent replicates, with error bars representing the standard deviation.

Figure S4

Figure S4: . ζ-Potential (mV) of lipid vesicles 1 and 5 ± Ca^2+^ at **(A)** pH 5.0, **(B)** pH 7.0, and **(C)** 8.0 as at various concentrations of daptomycin (µM). Data points represent averages of at least 3 independent replicates, with error bars representing the standard deviation.

Figure S5

Figure S5. Percent permeabilized *S. aureus* cells in buffer (20 mM Tris, 140 mM NaCl, 5 mM CaCl2) determined by propidium iodide fluorescence and flow cytometry.

| Table S1 | | | | | | | | | | | | |
| --- | --- | --- | --- | --- | --- | --- | --- | --- | --- | --- | --- | --- |
| Vesicle Sizes by DLS (nm) | | | | | | | | | | | | |
| Vesicle Composition | pH 4 - Ca^2+^ | | | pH 4 + Ca^2+^ | | | pH 8 - Ca^2+^ | | | pH 8 + Ca^2+^ | | |
| LV 1 | 167 | + | 55 | 167 | + | 27 | 182 | + | 43 | 127 | + | 19 |
| LV 2 | 112 | + | 6 | 141 | + | 31 | 178 | + | 63 | 202 | + | 154 |
| LV 3 | 172 | + | 36 | 163 | + | 30 | 162 | + | 14 | 154 | + | 48 |
| LV 4 | 139 | + | 12 | 163 | + | 30 | 144 | + | 47 | 144 | + | 47 |
| LV 5 | 155 | + | 26 | 127 | + | 19 | 135 | + | 32 | 202 | + | 83 |
